# Endocrine Disruption of the Follicle-Stimulating Hormone Receptor Signaling During the Human Antral Follicle Growth

**DOI:** 10.3389/fendo.2021.791763

**Published:** 2021-12-08

**Authors:** Neena Roy, Elisa Mascolo, Clara Lazzaretti, Elia Paradiso, Sara D’Alessandro, Kornelia Zaręba, Manuela Simoni, Livio Casarini

**Affiliations:** ^1^ Unit of Endocrinology, Department of Biomedical, Metabolic and Neural Sciences, Ospedale Civile Sant’Agostino-Estense, University of Modena and Reggio Emilia, Modena, Italy; ^2^ International PhD School in Clinical and Experimental Medicine (CEM), University of Modena and Reggio Emilia, Modena, Italy; ^3^ First Department of Obstetrics and Gynecology, Center of Postgraduate Medical Education, Warsaw, Poland; ^4^ Center for Genomic Research, University of Modena and Reggio Emilia, Modena, Italy; ^5^ Unit of Endocrinology, Department of Medical Specialties, Azienda Ospedaliero-Universitaria di Modena, Modena, Italy

**Keywords:** FSHR, FSH, endocrine disruptors, LHCGR, GPER

## Abstract

An increasing number of pollutants with endocrine disrupting potential are accumulating in the environment, increasing the exposure risk for humans. Several of them are known or suspected to interfere with endocrine signals, impairing reproductive functions. Follicle-stimulating hormone (FSH) is a glycoprotein playing an essential role in supporting antral follicle maturation and may be a target of disrupting chemicals (EDs) likely impacting female fertility. EDs may interfere with FSH-mediated signals at different levels, since they may modulate the mRNA or protein levels of both the hormone and its receptor (FSHR), perturb the functioning of partner membrane molecules, modify intracellular signal transduction pathways and gene expression. *In vitro* studies and animal models provided results helpful to understand ED modes of action and suggest that they could effectively play a role as molecules interfering with the female reproductive system. However, most of these data are potentially subjected to experimental limitations and need to be confirmed by long-term observations in human.

## Introduction

An increasing number of organic pollutants are accumulating in wastewater and soil. They may not necessarily be eliminated by purification treatments and could be potentially damaging for animals, as well as for human health. This issue has been taken into serious consideration by some Countries, such as in the European Union (EU), which issued specific directives to monitor and limit the impact of pollutants (1). The most common of these compounds are known to interfere with endocrine functions, such as estrogen signaling (2, 3). However, less is known about the potential impact of pollutants on gonadotropins’ receptor functions. These receptors regulate development and reproduction, and are potential targets for disrupting chemicals that negatively impact human health. In fact, several molecules targeting gonadotropin receptors possessing agonistic, antagonistic, or inverse agonistic activities, were tested *in vitro* and described (4).

Follicle-stimulating hormone (FSH) is a gonadotropin produced by the pituitary gland which binds its receptor (FSHR) located in the gonads (5). In men, FSHR is expressed in Sertoli cells, which provide physical support to spermatogenesis, in concert with the growth signal delivered by luteinizing hormone (LH) through the production of testosterone (6). In women, FSHR is co-expressed with the LH receptor (LHCGR) in ovarian granulosa cells, which is where androgen conversion to estrogens occurs and which support follicle selection, growth, and maturation during the antral stage of the menstrual cycle (7). These effects are accompanied by extremely dynamic variations of the FSHR number occurring throughout the follicular antral stage (8). As a result, the receptor achieves maximal expression levels in the early antral follicle, while decreasing with the progression of dominant follicle selection and maturation. In the preovulatory follicle, FSHR expression is almost entirely replaced by LHCGR, which is required for ovulation.

## Overview of FSHR Structure and Antral Stage-Specific Signaling

**Figure 1:**
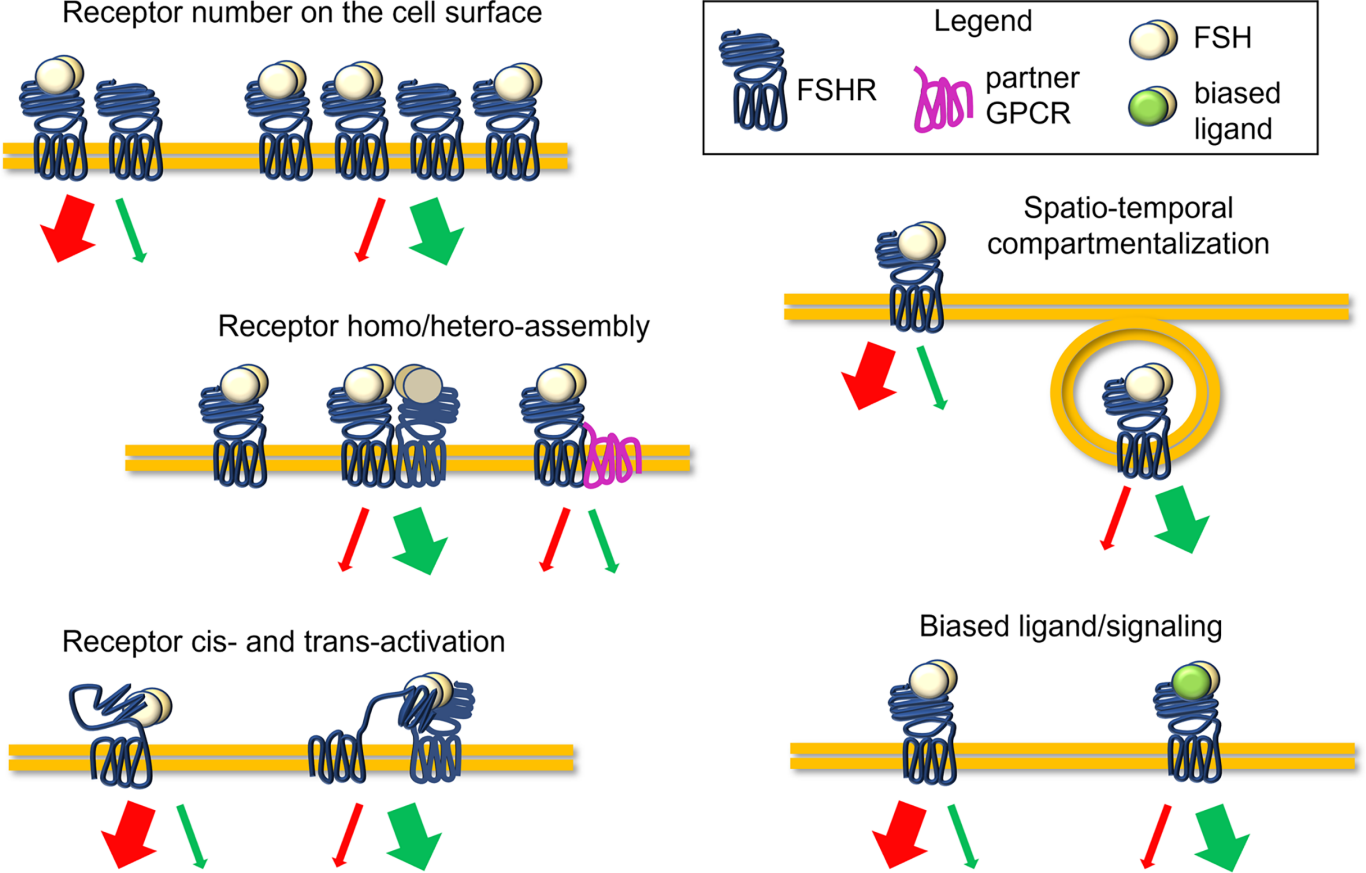
Complexity of the FSHR signaling regulation. Molecules with disrupting activity might potentially interfere with the FSH-induced signaling, impacting FSHR expression levels, receptor conformational assembly and cis/trans-activation, the compartmentalization of ligand-receptor complexes, and biased signaling, acting as allosteric modulators. Finally, disrupting molecules may modulate serum FSH levels or hormone binding to the receptor.

FSHR belongs to the subfamily of the rhodopsin-like G-protein coupled receptors, as the other glycoprotein hormone receptors to which it is structurally similar (5). The receptor is composed of a large NH_2_-terminal extracellular domain (ECD), embedding the hormone binding site, and is connected to the transmembrane domain (TMD) through a hinge region. The TMD passes through the cell membrane with seven *α* helices connected by alternating extracellular and intracellular loops (9). The latter, together with the C-tail in the intracellular side, carries interaction sites for G proteins and other transducing partners (9, 10). The ligand steric hindrance induces conformational changes of ECD, hinge region, and TMD, triggering a complex network of signaling cascades converging in proliferative, steroidogenic, pro-, and anti-apoptotic signals (4, 10). Receptor bound to the ligand may itself activate (cis-activation) these signals, as well as transducing the signal to activate other non-liganded receptors (trans-activation) (11). The action of FSH was classically associated with the activation of the steroidogenic G_α_s protein/cAMP/protein kinase A (PKA)-pathway, resulting in the transcription of several genes, such as those encoding steroidogenic enzymes (4). Together with steroidogenesis, this classical pathway can induce the activation of several other events that can lead at the same time to mitogenic signals, cytoskeletal changes, and apoptosis by stimulating the activation of other effectors (10). In fact, the FSH-dependent steroidogenic signalling pathway is counterintuitively linked to pro-apoptotic cascades which relies on p38 mitogen-activated protein kinase (MAPK) activation (12). Simultaneously, cAMP induces the activation of steroidogenic, anti-apoptotic and proliferative events mediated by extracellularly-regulated kinases 1 and 2 (ERK1/2) (10, 12). Despite these events must be fully clarified and investigated, they suggest a possible molecular mechanism underlying follicular growth and selection which may depend on the potency and persistence of cAMP at the intracellular level (10). G_α_i and Gq/11 proteins were also demonstrated to be coupled to FSHR, inducing respectively ERK1/2 phosphorylation, and phospholipase C (PLC) activation and intracellular calcium ion (Ca^2+^) increase (10, 13). Additional pathways and molecular partners of FSHR activation were described, such as insulin growth factor 1 receptor (IGF-1R), epidermal growth factor receptor (EGFR) (14, 15), 14-3-3τ protein (16, 17) and forkhead-box transcription factor O (FOXO1a) (18). Moreover, the molecules β-arrestins (19, 20), adaptor protein containing pleckstrin homology domain, phosphotyrosine binding domain, and leucine zipper motif (APPL1), are involved in the internalization and recycling of the receptor (21, 22). Recently, several studies demonstrated how the predominance of steroidogenic and pro-apoptotic, rather than proliferative signals, could depend on FSHR expression levels on the cell surface (20). While a relatively low number of FSHR on the membrane results in the preferential β-arrestins recruitment and pERK1/2 activation, relatively high FSHR expression levels lead to persistent intracellular cAMP accumulation linked to caspase 3 cleavage and apoptosis (20, 23). β-arrestins play a key role in inducing the FSHR internalization, an event fundamental for routing the hormone-receptor complex to recycling or lysosomal degradation pathways through specific endosomes (24–26). While these mechanisms may provide further examples of the complexity of FSHR regulation (**Figure 1**), they may be relevant to the selection of the dominant follicle, when serum FSH and other hormone levels change in a follicle and stage-dependent manner (7, 8, 10).

In women, disruption of FSH signaling is linked to infertility (27). These data match those from studies in Fshr-deficient mice, which were sterile due to failure of follicle maturation (28–30). Interestingly, this phenotype is not completely like that observed in FSHβ knock-out (KO) mice, which instead displayed a higher number of ovarian pathologies (31). This finding is suggestive of a dysregulated, but existing basal stimulation of the FSHβ KO mice ovary operated by intact receptors, which are absent in Fshr-deficient mice. However, these mice were characterized by relatively high serum LH levels. It is plausible that the chronic exposure to high concentrations of LH would be linked to the observed pathology *via* cross-interaction between FSHR and the hormone. These data are indicative of the relevance of proper FSH receptor functioning for ovarian physiology.

## Membrane GPCR Partners of FSHR

The activation of FSHR-mediated intracellular signaling pathways is further modulated by the presence of partner proteins located in the cell membrane, which may form heteromeric complexes perturbing FSH-dependent activity (32, 33). The expression levels of these molecules may vary during developmental stages of the follicle (4). FSHR, as many other GPCRs, is demonstrated to form homo- and heteromeric complexes on the cell surface with other receptors with similar protein structure (32–34). For instance, the presence of FSHR/LHCGR complexes was reported when overexpressed in transiently transfected cells (11, 33, 34) and genetically modified mice (35). After binding LH, LHCGR independently activates two G protein-dependent signaling pathways, adenylyl cyclase, activating cAMP and the downstream cAMP-response element binding protein (CREB), ERK1/2 mainly *via* G_α_i protein and β-arrestins, and PLC for Ca^2+^ mobilization from intracellular stores (36, 37). Gq- or βγ-dependent phosphatidylinositol-3-kinase (PI3K)/protein kinase B (AKT)-pathway activation occurs simultaneously, promoting cell survival and growth. LHCGR target genes, such as the steroidogenic acute regulatory protein (STARD1), mediate steroidogenic and anti-apoptotic/proliferative events in granulosa cells (38). At the intracellular level, the interaction between the two receptors would result in the modulation of the LH-induced signaling, occurring in the presence of FSH, which results in the potentiation of anti-apoptotic signals (39) and receptor-receptor interactions (40). Finally, the interaction between FSHR and LHCGR is linked to abolition of Ca^2+^ responses, due to rearrangements of the receptor structure impairing Gq protein associations (32). Therefore, LHCGR is a known membrane FSHR partner, in granulosa cells, impacting the FSH-specific signals.

More recently, FSHR was demonstrated to interact with the G protein-coupled estrogen receptor (GPER). FSHR/GPER complexes would be present on the surface of primary granulosa cells, where they inhibit the intracellular cAMP accumulation and stimulate proliferative signals activating the AKT pathway upon FSH binding (4, 41). The balance between cAMP- and AKT-dependent events would play a key role in determining the fate of the ovarian follicle, which could be addressed to dominance or *atresia*, and is linked to the responsiveness to controlled ovarian stimulation treatments (41). Taken together, those findings add new insights in the understanding of the crosstalk between signalling cascades and suggest that FSHR signaling may be modulated by disrupting compounds targeting membrane partners co-expressed during the antral stage.

## Main Factors Disrupting Reproductive Functions

Over the years, certain chemicals or compounds, either natural or synthetic, present in the environment have been identified to disrupt the endocrine function of the reproductive system. Such compounds are termed endocrine disruptors (EDs) and may disrupt normal homeostatic endocrine function. Additionally, certain compounds bind to a site distinct from the ligand binding site and impacts receptor signalling, acting as allosteric modulators. These molecules can bias, positively or negatively the signal transduction pathways linked to ovarian receptors. The mode of action of most of the EDs or allosteric modulators has not been clearly determined, and more studies are still required to fathom their effect on female reproductive health. According to a recent consensus statement (42), an ED may have the following ten characteristics: 1) interacts with or activates hormone receptors, 2) antagonizes hormone receptors, 3) alters hormone receptor expression, 4) alters signal transduction in hormone-responsive cells, 5) induces epigenetic modifications in hormone-producing or hormone-responsive cells, 6) alters hormone synthesis, 7) alters hormone transport across cell membranes, 8) alters hormone distribution or circulating levels of hormones, 9) alters hormone metabolism or clearance and 10) alters the fate of hormone-producing or hormone-responsive cells. EDs include natural compounds such as phytoestrogens (e,g., genistein and coumestrol), polycyclic aromatic hydrocarbons [benzo(a)pyrene] and synthetic chemicals used as industrial solvents/lubricants and their byproducts [polychlorinated biphenyls (PCBs), polybrominated biphenyls (PBBs), dioxins], plastics [bisphenol A (BPA)], plasticizers (phthalates), pesticides [methoxychlor, dichlorodiphenyltrichloroethane (DDT)], fungicides (vinclozolin), and pharmaceutical agents [diethylstilbestrol (DES)]. EDs such as pesticides and insecticides, factory smoke and household dust (43), access human through air by means of aerosol spray, contaminated water and through food by means of leaching of these chemicals from packaging. Moreover, EDs may come from consumption of animals, fish or plants exposed to these chemicals (44–47). Despite different route of exposure, EDs affect both men and women of different age leading to detrimental reproductive outcomes including infertility, endometriosis, and polycystic ovarian syndrome (PCOS) (48, 49). Although some chemicals are banned because of recognized ED effect, they still persist in the environment and have different effects on reproductive health, depending if the exposure occurs at the prenatal, perinatal, or postnatal age (50). They are known to affect steroidogenesis and folliculogenesis (51), thereby causing infertility, poor implantation and interference with placental functions (52–54). The exact mechanism of their mode of action is often unknown, however, the regulation of ovarian antral follicles by gonadotropins could be a site not to be overlooked. Due to the scarcity of available literature on the mechanism of binding of the ED, it is imperative to discuss this in the light of allosteric modulators. Allosteric modulators are thought to bind to the TMD of the receptor. Very recently, a study unravelled the cryo-electron microscopy structure of full-length LHCGR, revealing a ‘push-and-pull’ mechanism for the LHCGR activation, i.e., the ECD is pushed by the bound hCG and pulled by the hinge loop next to TMD (55). When Org43553, an allosteric agonist was used, it bound to a pocket of the TMD and interacted with a highly conserved 10-residue fragment (P10), thereby stabilizing the active conformation (55). While, LUF5771, an allosteric LHCGR inhibitor, interacted directly with hydrophobic aminoacids in the minor pocket formed between transmembrane helices 1-2 and 7, which restricted the receptor to a more inactive conformation (56). Perhaps, these studies could be used as a common model for understanding the structure-function relationship of FSHR with their agonist or antagonists. In fact, the crystal structure of FSH bound to FSHR ECD revealed that the FSH binding to the inner concave surface of leucine-rich repeats (LRR), present in ECD, exposes the sTyr-binding pocket in the FSH ligand. Following this, the FSHR inserts its sTyr into the FSH nascent pocket, thereby activating the receptor (57). ADX68692, an allosteric inhibitor of FSHR, was proposed to act by disturbing TMD and thereby to open up the FSHR trimer to allow binding of two additional FSH as they suggested that FSHR trimer could bind to only one FSH to engage G protein and subsequent activation of adenylate cyclase (58). Since the allosteric site is highly conserved in the other glycoprotein hormone receptors like LHR and TSHR, the final effects on the FSH-dependent follicular growth could be indirectly mediated through these other receptors as well. Possibly, these mechanisms could throw some light on the mechanism of interaction of ED with FSHR.

## Impact of EDs on FSHR Expression, Functioning, and Signaling

**Figure 2:**
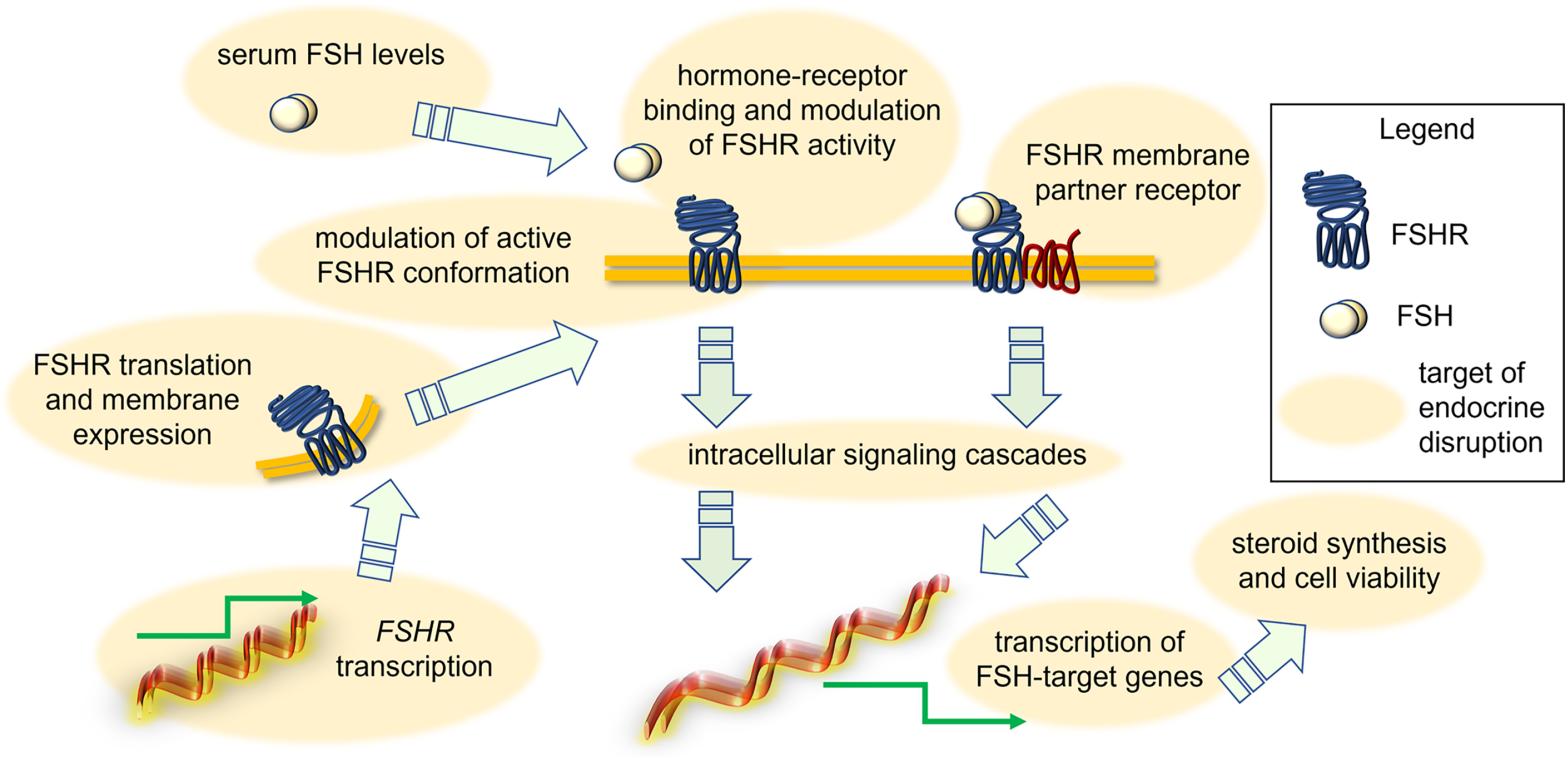
Action of EDs in disrupting FSH-dependent endocrine signals in the ovary. Depending on the type of EDs, the FSH signal may be modulated due to changes of FSH levels, FSHR expression and attenuation of active conformation, perturbation of interaction with membrane GPCR partners, intracellular signaling cascades and target gene expression, synthesis of steroids and granulosa cell viability.

It was suggested that some EDs act through a direct effect on *FSHR* transcription, protein-mediated intracellular signaling (**Figure 2**). An example is provided by 1-chloro-4-[2,2,2-trichloro-1-(4-chlorophenyl)ethyl]benzene (p,p’-DDT), which interacts with a specific amino acid portion of FSHR, thereby modifying the physiochemical environment of TMD of the receptor and acting as a positive allosteric modulator. Also, the binding of p,p’-DDT released the inhibitory interaction of ectodomain to TMD of the receptor to increase the sensitivity of the receptor to human chorionic gonadotropin (hCG), which it is not its canonical ligand (59). This study also confirms the idea that the main target of ED is receptor itself and thereby mediating its effect. In another study, DDT exerted its inhibitory effect on cAMP accumulation in thyroid-stimulating hormone receptor (TSHR)-expressing Chinese hamnster ovary (CHO) cells by inhibiting the constitutive activity of TSHR and not due to any other downstream effectors like Gαs protein (60). However, other molecular mechanisms of endocrine disruption were found. For instance, exposure to benzene, a major product from tobacco smoking, automobile service stations, exhaust from motor vehicles and industrial emissions, has been shown to negatively impact the ovarian function in *in vitro* fertilization (IVF) settings (61). It adversely affected ovarian response to exogeneous gonadotropins, reducing follicular reserve *via* modulation of the transduction efficiency of *FSHR* (61). Another example is provided by the *in vitro* effect of carbon-black nanoparticles, where these inhibited FSH-induced aromatase expression and activity in the KGN human granulosa cell line (62). A different mechanism of action seems to involve the endocrine disruption caused by mono(2-ethylhexyl) phthalate (MEHP), the active metabolite of di(2-ethylhexyl) phthalate (DEHP). It decreased FSH-induced cAMP accumulation in rat granulosa cells (63), suggesting that this ED impacts directly FSHR functions or, at least, the early signaling of the receptor. These effects have repercussions on FSH-induced progesterone production, which is inhibited by MEHP likely *via* a protein kinase-C independent mechanism (64). In contrast, a study in rat ovarian granulosa cells *in vitro* showed that MEHP (25-100 µM) inhibited granulosa cell proliferation, upregulated expression of sex steroid receptors and key enzymes in progesterone production, finally resulting in increased progesterone and estradiol synthesis (65). Therefore, MEHP seems to modulate “species-specific” effects which might rely on differences of receptor structure, as well as on other ovarian molecular targets and hormonal *milieu*. In any case, given that MEHP impacts the role of sex steroids fundamental to support follicular maturation, we may speculate that accumulation of this compound in the environment requires attention to preserve human health. Moreover, monitoring of the MEHP precursor DEHP is worth attention as well. Prolonged exposures to a lower dose (0.05mg/kg/day) of DEHP resulted in reduced expression of *Cyp17a1*, *Cyp19a1*, *progesterone receptor (Pgr), Lhcgr* and *Fshr* in the adult ovary (PND41) of CD-1 mice, affecting ovarian steroidogenesis (66). Interestingly, short-term exposure to DEHP increased FSH at multiple doses until 6 months post-dosing, likely consisting in the compensatory feedback mechanism due to low *Fshr* expression and subsequent insufficient follicular response to physiological FSH levels (67). Similar results were obtained upon treatment of female mice with chlorothalonil, a fungicide used in horticulture (68). In any case, the DEHP mechanism of action seems to be opposite, or anyway different to that of the dimethyl phthalate (DMP). Indeed, long term exposure to DMP is linked to decreased secretion of FSH and increased secretion of estradiol and LH in C57 female mice. DMP also interferes with the pituitary-ovary axis and increased the apoptosis rate of ovarian granulosa cells (69). The treatment with dibutyl phthalate (DBP) (0.1 mg/kg/day) increased FSH production even in CD-1 mice, reducing the antral follicle number and increasing mRNA level of pro-apoptotic genes, such as *Bax*, *Bad* and *Bid* (70). *In vitro* studies attempted to elucidate the DBP mechanism of action. For instance, in the tumour cell line KGN, 24-h treatment with 0.1 µM DBP upregulated *FSHR* mRNA, as well as *CYP19A1* mRNA and protein, and increased estradiol production (71). In rat granulosa cells and preantral follicles treated by DBP *in vitro*, FSH failed to induce KIT ligand mRNA and protein expression, and steroidogenesis, due to FSHR downregulation (72). Thus, experiments using DBP strengthened the hypothesis that EDs modulate species-specific effects.

Interestingly, some studies tested the effects of a mixture of different EDs. For instance, a combination of three phthalates [bis (2-ethylhexyl), dibutyl, and benzyl butyl] and two alkylphenols (4-nonylphenol and 4-tert-octylphenol) decreased both mRNA and protein expression of *Fshr*, *Lhr*, and *Cyp19a1* in female mice, at 10 mg/kg/d. These changes resulted in altered steroidogenesis and loss of antral follicles (73), suggesting that the exposure to multiple EDs may be linked to cumulative, disruptive actions with effects even more deleterious than those of one single compound.

One of the most studied ED is the BPA, which is known to interfere with the estrogen signaling and to suppress ovarian function in aquatic animals, such as the zebrafish (74). In this case, the endocrine disruptive effect is suggested to rely on interference with estrogens and LH receptor-controlled gene expression (75). However, exposure to environmental-relevant levels of BPA has been shown to alter steroidogenesis and downregulated the expression of Gs protein suggesting the suppression of the FSHR/G_α_s protein/adenylyl cyclase signaling pathway, in human granulosa cell lines (76). BPA was also shown to downregulate FSH-stimulated insulin-like growth factor 1 (IGF-1), steroidogenic factor-1 (SF-1), GATA4, aromatase, and estradiol in human granulosa cells by upregulating the expression of peroxisome proliferator-activated receptor-gamma (PPARγ) (77). *In vivo* data from pregnant female rats also revealed the potential impact of BPA on the foetus (78). Offspring born from mothers perinatally exposed to this molecule had impaired ovarian response to gonadotropins. Offspring treated with gonadotropins, i.e. pregnant mare serum gonadotropins (PMSG) and human choriogonadotropin (hCG) developed persistent, high *Fshr* mRNA expression. As a result, the ovarian follicles of these mice had a decreased number of follicles, during the antral stage, and they were characterized by relatively high expression of the progesterone receptor. After ovulation, there was an increase in antral atretic follicles, reduced *Lhr* mRNA expression and high serum levels of estradiol (78). Taken together, these studies suggested that BPA may directly impact FSHR-mediated signals. However, recent data revealing the molecular basis by which BPA may bind GPER (79) are suggestive of modulatory effects of FSH signals *via* targeting of FSHR membrane partners. In fact, BPA induced KGN granulosa cell death *via* GPER-mediated activation of reactive oxygen species (ROS) and intracellular calcium Ca^2+^ increase, *in vitro* (80). Given that GPER and FSHR may cooperate to support follicle selection and maturation during the antral stage (41), these data suggest that FSHR-mediated intracellular signaling might be perturbed upon disruption of GPER action. Similarly, in chickens, it was demonstrated that 4-Nonylphenol (4-NP) downregulated the ovarian expression of both FSHR and LHR, while it upregulated the levels of steroidogenic enzymes and of the estrogen receptor alpha (erα) (81). Again, these data may lead to the hypothesis that the disruptive effect of some EDs could be due to the action on FSHR membrane partners, although physical interactions between gonadotropin receptors were never demonstrated in chicken ovaries.

Although some ED mechanisms of action are well-defined, there are experimental limitations preventing conclusive assumptions. First, long treatment time is required to provide useful information, but it is not necessarily possible to set properly long *in vitro* experiment. Moreover, in a large part of cases, specimens used for *in vivo* experiments do not provide results translatable to human. Finally, real control groups unexposed to EDs were not possible due to the presence of interfering molecules in laboratory plastics and in the environment (82).

## ED’s Effects Indirectly Related to FSHR

**Figure 3:**
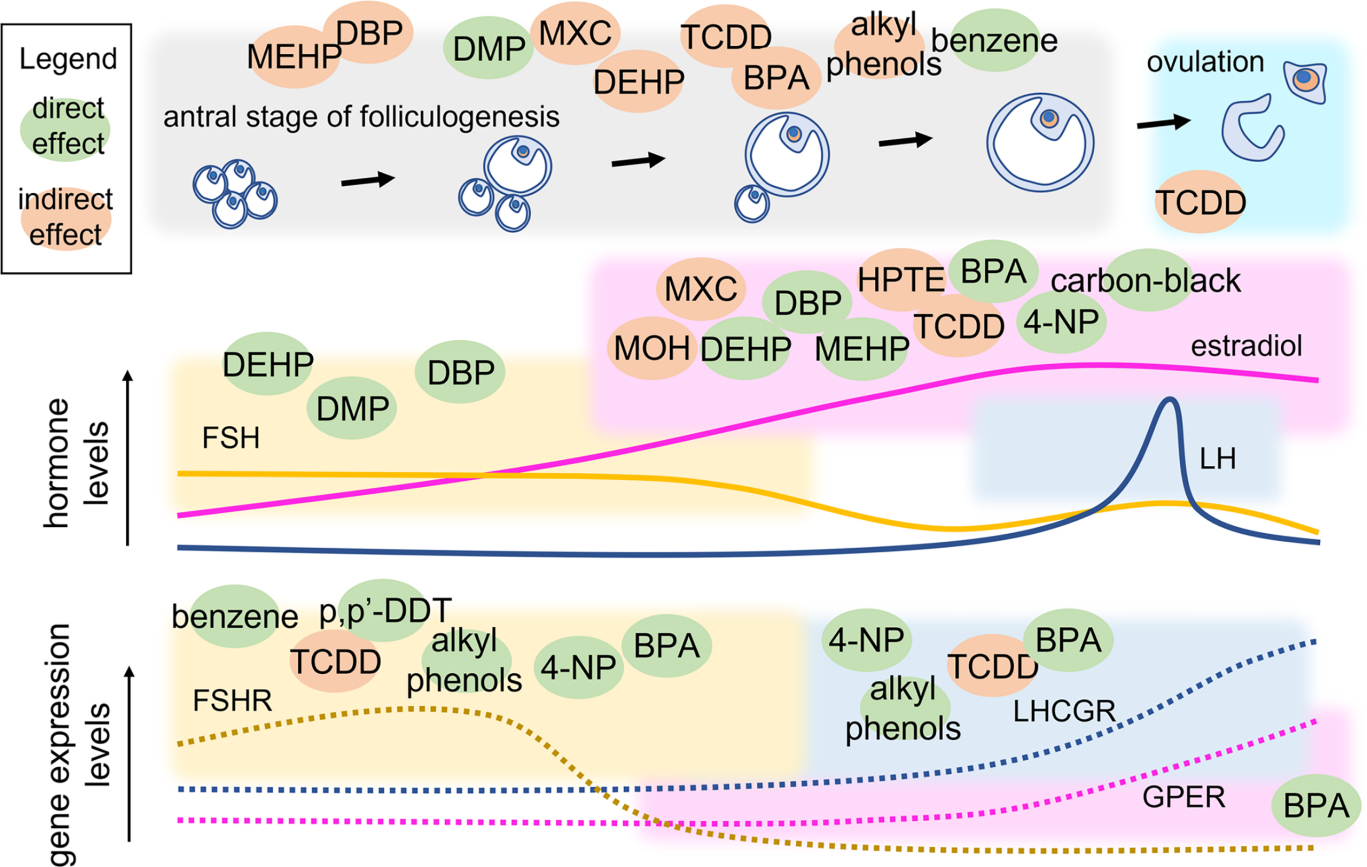
Point of action of EDs in the antral stage of folliculogenesis. Variations of hormone and receptor expression levels are shown together with follicle growth. EDs are located within coloured squares indicating the endpoint that they may modulate directly (green) or indirectly (orange). MEHP, mono(2-ethylhexyl) phthalate; DEHP, di(2-ethylhexyl) phthalate; DBP, dibutyl phthalate; DMP, dimethyl phthalate; MXC, Methoxychlor; TCDD, 2,3,7,8-Tetrachlorodibenzo-p-Dioxin; BPA, bisphenol A; 4-NP, 4-Nonylphenol; p’p-DDT, methoxychlor, dichlorodiphenyltrichloroethane; MOH, 1,1,1-trichloro-2-(4-hydroxyphenyl)-2-(4-methoxyphenyl) ethane; HPTE, 1,1,1-trichloro-2,2-bis(4-hydroxyphenyl).

Antral folliculogenesis is regulated by various endocrine and paracrine factors, including gonadotropins (83). Therefore, disruption of antral folliculogenesis (**Figure 3**) is suggestive of a possible link with infertility. It is known that different EDs can affect antral folliculogenesis (84). BPA, a well-studied ED whose primary source is diet (85, 86), has been shown to affect antral folliculogenesis. In a study conducted on adult female rats exposed to BPA (25 ng/kg/d or 5 mg/kg/d) for 15 days, a reversible decrease in antral follicles and *corpora lutea* was noted, likely inducing delay and decrease of the LH surge amplitude (87). BPA exposure of 28-week old rats for 42 days resulted in large, antral-like follicles and atretic, cystic-like follicles that did not reach ovulation stage (88). Using an *in-vitro* follicle culture system, it was found that BPA inhibited follicle growth and steroidogenesis in mouse ovarian antral follicles (89). In a later study, the same authors demonstrated that BPA likely reversibly targets genes coding steroidogenic enzymes, inhibiting steroid hormone production (90). At low concentration, BPA could induce epigenetic changes during follicle culture and oocyte growth that may affect health of the offspring (91). Moreover, exposure to BPA during the early postnatal period is linked to decreased methylation of *IGF2R* and *PEG3* imprinted genes and suppresses the expression of DNA methylation transferases, which were closely related to oocyte growth. In human, a link between BPA exposure and PCOS was found. In women with PCOS, an inverse association between urinary BPA concentration and antral follicle count was demonstrated, suggesting that BPA affects ovarian follicle and thereby will reduce ovarian reserve (92). BPA was found to be associated with women undergoing medically assisted reproduction as well. In such women, high urinary BPA levels were associated with low antral follicle count which could result in accelerated follicle loss (93). Finally, it was demonstrated that BPA may lead to kidney alterations (94, 95). These data support the concept by which BPA could extend the gonadotropins’ half-life by decreasing the renal glomerular filtration (96), thus exposing the organism to persisting FSH-mediated effects.

Other highly investigated EDs are phthalates. DEHP and MEHP are the most commonly used phthalate ester present in consumer products. Several studies have shown that both DEHP and MEHP affects female fertility by affecting antral follicle functionality. An *in vitro* study has demonstrated that DEHP and MEHP directly inhibit antral follicle growth *via* a mechanism that partially includes reduction in levels of estradiol production and decreased expression of cell cycle regulators (97). DEHP inhibits follicle growth possibly through dysregulation of the cell cycle, induces *atresia* likely *via* dysregulation of apoptosis, and inhibits steroidogenesis possibly due to lack of upstream sex steroid hormones and disruption of the steroidogenic enzymes (98). Exposure of female mice to DEHP has been shown to disrupt steriodogenic enzymes in F2 and F3 generations and altered DNA methylation in the ovaries (99, 100). In fact, it is known that EDs may alter the epigenetic profile (101), as a disrupting event that was described to occur at the level of non coding RNAs, such as the transfer RNA (tRNA) possibly impacting protein expression levels (102). Certain studies have used a mixture of chemical compounds to evaluate their conjoint effect on female reproductive health. In a study employing a mixture of phthalates (DEHP, dibutyl, and benzyl butyl) and alkyl phenols (4-nonylphenol and 4-tert-octylphenol) at an environmentally relevant dose, the authors demonstrated reproductive alterations in chronically exposed female mice. At the lowest dose (1-mg/kg BW/d), the mixture delayed the onset of puberty and the transition from preantral to antral follicles, whereas the highest dose used (10-mg/kg BW/d) decreased the number of antral follicles and gonadotropin receptor expression (73). Methoxychlor (MXC) is an organochlorine pesticide that affect female reproductive health and gains access to humans primarily through contaminated food and water (103). Adult female mice exposed to MXC showed ovarian atrophy due to inhibition of folliculogenesis leading to atretic follicles and reduced ovulation (104). Further studies have shown that MXC promotes antral follicle atresia in female mice (105, 106) and inhibits steroidogenesis (106, 107). MXC mainly acts through its metabolites, 1,1,1-trichloro-2-(4-hydroxyphenyl)-2-(4-methoxyphenyl) ethane (MOH) and the bisphenolic compound 1,1,1-trichloro-2,2-bis(4-hydroxyphenyl) ethane (HPTE). MOH inhibits steroidogenesis both by reducing the availability of pregnenolone (108) and by inhibiting the expression levels of key steroidogenic enzymes, *Cyp11a1*, *Cyp17a1*, and *Cyp19a1* mRNA in mouse antral follicles *in vitro* (109). Similarly, HPTE (1–10 μM) reduces FSH-stimulated synthesis of progesterone and estrogen by lowering the *Cyp11a1* and *Cyp19a1* mRNA in cultured rat granulosa cells (109). 2,3,7,8-Tetrachlorodibenzo-*p*-Dioxin (TCDD) belongs to a class of dioxins that disrupts folliculogenesis, steriodogenesis and ovulation. TCDD has an antiproliferative effect on the rat ovary as suggested by decreased number of antral follicles without increasing atresia on TCDD exposure (110). TCDD blocks ovulation in gonadotropin-primed immature rats by reducing the number of granulosa cells in S phase and inhibiting the levels of cyclin dependent kinase 2 (*Cdk2*) and *Ccnd2* (111). Another study in rat granulosa cells showed that TCDD (10 pM) suppresses the expression and mRNA stability of FSH-induced LH receptors, suggesting that TCDD disrupts the signaling pathway that responds to LH-induced ovulation (112). TCDD exposure also decreases ovarian steroidogenesis by inhibiting key steroidogenic enzymes (*Hsd17b1*and *Cyp19a1*), leading to reduced steroidogenic capacity of antral follicles (113).

Finally, several EDs target the ovary and affect folliculogenesis, ovulation and steroidogenesis, *via* effects indirectly targeting gonadotropin-dependent functions. These compounds impact folliculogenesis, ovulation and steroidogenesis, and may induce a long-lasting effect on reproductive, but also on non-reproductive health as these processes are important for the cardiovascular, skeletal and brain health. *In vitro* studies provided evidence, partially confirmed in animal models (114, 115), suggesting a possible disruptive effect of environmental pollutants on the antral stage. However, the impact on human folliculogenesis is still poorly known and could be elucidated by long-term epidemiological observations on large datasets. These data shed lights on EDs’ new modes of action. These compounds were classically described as molecules targeting the orthosteric site of nuclear hormone receptors, while recent studies suggested that they might bind a structurally similar allosteric site of other GPCRs (116). Therefore, screening methods based on molecular docking could be used for predicting potential, new EDs binding to FSHR.

## Conclusions

Environmental pollutants which cause endocrine disruption may impact FSHR signals at different levels. They may target direcly receptors expressed in the surface of ovarian cells, acting as allosteric modulators and binding promiscuity. Other compounds up- or downregulate receptor expression or are suggestive of FSH signal disruption altering signaling to FSHR membrane partner receptors, modulating their physical interaction or the crosstalk between intrracellular signaling pathways. These actions may negatively impact oocyte maturation, which occurs during the antral follicular stage accompanied by FSHR expression. Although evidence is accumulating over the last years, there are experimental limitations, such as the presence of EDs in laboratory plastics, to overcome before achieving a full dissection of molecular mechanisms at the basis of FSHR signal disruption. Further EDs could be discovered by docking studies evaluating potential interactions between these molecules and FSHR allosteric sites.

## Author Contributions

NR, EM, EP, CL, SD’A, and KZ: writing — original draft preparation. MS and LC: writing — review and editing. LC: visualization. MS and LC: supervision. LC: conceptualization. All authors have read and agreed to the published version of the manuscript.

## Funding

This work was supported by the Italian Ministry of University and Research (MUR) by “Progetti di Rilevante Interesse Nazionale” (PRIN2017).

## Conflict of Interest

The authors declare that the research was conducted in the absence of any commercial or financial relationships that could be construed as a potential conflict of interest.

## Publisher’s Note

All claims expressed in this article are solely those of the authors and do not necessarily represent those of their affiliated organizations, or those of the publisher, the editors and the reviewers. Any product that may be evaluated in this article, or claim that may be made by its manufacturer, is not guaranteed or endorsed by the publisher.
